# A Retrospective Forensic Review of Unexpected Infectious Deaths

**DOI:** 10.1093/ofid/ofz081

**Published:** 2019-02-15

**Authors:** Prateek Sehgal, Michael Pollanen, Nick Daneman

**Affiliations:** 1 Department of Medicine, Toronto, Canada; 3 Division of Infectious Diseases and Clinical Epidemiology, Sunnybrook Health Sciences Center, Toronto, Canada; 2 Ontario Forensic Pathology Service, Toronto, Canada

**Keywords:** epidemiology, forensic, infectious diseases, sepsis, unexpected death

## Abstract

**Background:**

There exists a knowledge gap in identifying the spectrum of infectious pathogens and syndromes that lead to fulminant decline and death. The aim of this study was to better characterize patient-, pathogen-, and disease-related factors in the phenomenon of unexpected infectious deaths.

**Methods:**

We conducted a population-based, retrospective cohort study of all community-based, unexpected infectious deaths in Ontario, Canada between January 2016 and December 2017. Patient-related information, infection-related information, and circumstances around the death were extracted for each case to facilitate descriptive analyses.

**Results:**

Of the 7506 unexpected deaths over the study period, 418 (6%) were due to infectious diseases. Bacterial pneumonia (43%) was the most common infectious syndrome, followed by disseminated infection with no clear focus (12%), peritonitis (10%), myocarditis (6%), and pyelonephritis (5%). A pathogen was identified in 210 cases (50%), with the most common being *Staphylococcus aureus* (n = 35), *Streptococcus pneumoniae* (n = 30), *Streptococcus pyogenes* (n = 25), *Klebsiella* spp. (n = 23), and *Escherichia coli* (n = 19). Prodromal symptoms were present in 68% of persons before death, with a median (interquartile range) duration of only 1.0 (0.0–4.0) days; just 30% of those who died had had recent healthcare contact before their death.

**Conclusion:**

Infectious diseases have the capacity to cause fulminant decline and death. The most common cause of unexpected infectious death is bacterial pneumonia, with a predominance of gram-positive bacteria. Given the rapidity of these deaths, preventing a majority of them would require upstream strategies to reduce infection susceptibility and transmission.

## Teaser

This population-wide cohort study highlights the phenomenon of unexpected deaths due to infectious disease, with respect to the key pathogens involved, the most prominent clinical syndromes, characteristics of those who died, and opportunities and challenges for prevention.

Mortality rates related to infectious disease in North America have decreased over the years owing to multiple factors, including the advent of improved public health and hygiene, vaccine and antimicrobial development, advances in supportive care for critically ill patients, and a better understanding of clinical infectious disease [1, 2]. However, there remains a subset of the population in whom health declines abruptly and unexpectedly owing to infectious pathogens. Such rapid decline may be related to the primary infectious process directly or indirectly through a secondary mechanism, as exemplified by recent evidence of an association between respiratory viral infections and myocardial infarctions [3].

The link between certain pathogens and rapid clinical decline has been well described previously for syndromes such as *Staphylococcus aureus* and group A streptococcal toxic shock, which are associated with significant mortality and morbidity rates [4]. However, most literature on fulminant infection derives from case series of patients admitted to hospitals or critical care units [5, 6]. There remains a knowledge gap in identifying the spectrum of pathogens and clinical syndromes that may lead to similar fulminant decline and death even before exposure to a healthcare setting. The phenomenon of unexpected death has largely been characterized for primary cardiac death or abrupt respiratory decline (eg, pulmonary embolism). The literature on unexpected deaths due to infectious disease has been sparse, particularly at the population level.

The overarching aim of this retrospective, population-based forensic study was to better characterize patient-, pathogen-, and disease-related factors in patients with unexpected infectious deaths across a broad population. By capturing all unexpected infectious deaths in the Province of Ontario, Canada, in 2016 and 2017, our specific aims were to profile the most common clinical pathogens and syndromes associated with unexpected infectious deaths, identify patient risk factors associated with them, and assess their potential preventability by examining the time course of clinical decline and healthcare system contact.

## MATERIALS AND METHODS

### General Study Design

Our study was designed as a population-based, retrospective cohort study in Ontario, Canada between 1 January 2016 and 31 December 2017. Ontario is Canada’s most populous province, with 14 139 384 residents in 2017 [7]. All data was obtained through coroner autopsies performed for unexpected deaths. Ethical approval was obtained from the Ontario Forensics Pathology Service (OFPS).

### Forensic Data Collection

Autopsy records were extracted from OFPS, which encompasses a catchment population including the provinces of Ontario and northeastern Manitoba in Canada. Autopsies were carried out for deaths that were considered traumatic, suspicious, pediatric, or unexpected in nature. Each case was assessed by a coroner who identified the circumstances surrounding the death along with the appropriateness and need for an autopsy investigation. All autopsies were carried out with supporting histological and microbiological investigations, as indicated based on the circumstances of death.

### Identification of Unexpected Infectious Deaths

Over the 2-year period, we identified coroner cases that were deemed to be unexpected. These cases included deaths that were pronounced on the scene at a decedent’s residence, en route to the hospital, or after recent admission to a hospital. Based on the final cause of death as determined by the pathologist, we identified cases in which the primary cause of death was an infectious disease.

Exclusion criteria excluded deaths in neonates associated with placental rather than independently acquired infection, deaths caused by pneumonia attributed to likely aspiration events, coroner findings suggestive of prolonged death with unknown time and acuity of death (eg, body showing advanced signs of decomposition), hospital-onset infections with subsequent death during the initial hospital stay, and deaths in which substance use was the primary cause, with associated infection found to be a minor contributing pathological factor.

### Definitions

Given the breadth of infection diagnoses, we attempted to group together clinically similar causes to simplify and focus the data. “Abscess” included cases in which a source of infection predominantly originated from an identifiable, single abscess, regardless of location in the body. Lobar pneumonias and bronchopneumonias were grouped together into “bacterial pneumonia,” and pneumonias with histological evidence of lower respiratory infection attributable to a viral pathogen were classified as “viral pneumonia.” “Myocarditis” included any cases of nonrheumatic myocarditis in which the suspected cause was infectious or idiopathic. The rationale for this was that pathogen specimens extracted from myocardial samples were generally low yield, but the histological samples in nonrheumatic myocarditis showed indirect findings consistent with recent viral infection. “Biliary infection” encompassed cases of cholecystitis, cholangitis, and gallstone pancreatitis. “Peritonitis” was defined as histopathological or gross anatomical evidence of peritoneal infection that was probably due to an intra-abdominal source, including ulcer perforation, small- or large-bowel perforation, pelvic inflammatory disease, or spontaneous bacterial peritonitis. “Disseminated infection with no clear focus” was defined as the presence of bacteria in multiple tissues without an evident source of primary infection.

“Time from most recent healthcare contact” and “prodrome length” were both measured in days. Prodromes were defined as a constellation of symptoms that predated the time of death but were probably attributable to the infection. Occasionally, coroner assessments reported nonspecific lengths of recent healthcare contact or prodrome using the terms “several days” or “few days.” These were categorized and displayed separately in our data analysis and not included in the calculation of summary descriptive statistics.

### Data Collection

To establish trends and identify potential risk factors for unexpected infectious death, we collected patient-related information, infection-related information, and circumstances around death for each unexpected infectious death. The patient-related variables analyzed included age, sex, comorbid conditions, and history of active or chronic substance use. This information was acquired through coroner histories, hospital records provided within the coroner report, or histopathological evidence of organ disease on review of prepared slides by pathologists. The representative comorbid conditions selected were hypertensive or atherosclerotic heart disease, diabetes mellitus, kidney disease, liver disease, lung disease, active cancer, and immunosuppression status (human immunodeficiency virus, chemotherapy, or other immunosuppressive medications).

With respect to infection-related factors, we identified antemortem and postmortem cultures (if sent and cultured), type of postmortem culture (blood or tissue), and identification of the clinical syndrome associated with death. Postmortem cultures were sent for investigation at the discretion of the attending pathologist. Given the propensity for postmortem cultures to have significant bacterial overgrowth, only cases in which the pathologist was confident that culture results correlated with true infection were included as true-positives. If 2 microbes were cultured and were both deemed clinically relevant, these were both included in the pathogen analyses. If >2 relevant microbes were identified, the cause was deemed to be “polymicrobial.”

Factors related to the circumstances of the death included location of death, presence of concurrent acute substance misuse, presence of prodromal symptoms and length of prodrome, and timing of most recent contact with the healthcare system before death. Given privacy limitations, we had access to the information only as documented within the coroner records, with no access to primary healthcare records unless integrated within the coroner office reports. Therefore, we did not have access to information about premortem community antibiotic use and the specific nature or outcome of prior healthcare visits.

### Data Analysis

We compared basic demographic and cause-of-death analyses between unexpected infectious deaths and noninfectious deaths through the OFPS database. We compared discrete variables using the χ^2^ test and continuous variables using the Mann-Whitney *U* test. The population incidence of unexpected infectious death was calculated by dividing the number of cases in 2016 and 2017 by Ontario population estimates for those years obtained from Statistics Canada [8]. The distribution of variables were summarized using medians and interquartile ranges (IQRs). We used SPSS software (version 22.0) for statistical analysis.

## RESULTS

### Patient-Related Factors

We were able to identify 7506 cases of unexpected death ascertained by autopsy over the 2-year period of the study. Of these, 418 (6%) were determined to be deaths due to infectious disease. Based on Ontario population estimates from the study period, there were 53 unexpected deaths and 2.9 unexpected infectious deaths per 100 000. The median (IQR) age of persons with unexpected infectious deaths was 55 (45–64) years, which did not differ significantly from that in persons with unexpected noninfectious deaths (54 [41–64] years; *P* = .27) [Table 1]. 

**Table 1.** Comparison of Unexpected Infectious and Noninfectious Deaths

Variable	Unexpected Deaths, No. (%)^a^		*P* Value
	Infectious (n = 418)	Noninfectious (n = 7088)	
Age, y			
Median	55 (45–64)	54 (41–64)	.27
Interquartile range	45–64	41–64	
Range	0–97	0–103	
Male sex, %	60	68	.001
Primary cause of death			
Respiratory	213 (51.0)	635 (9.0)	<.001
Abdominal	80 (19.1)	295 (4.1)	<.001
Other	70 (16.7)	3659 (51.7)	<.001
Cardiac	42 (10.0)	2213 (31.2)	<.001
Neurological	13 (3.1)	286 (4.0)	.35

^a^Data represent No. (%) of deaths, unless otherwise specified.

There was a male predominance in both groups, but the sex difference was less pronounced in unexpected infectious deaths with males representing 60% of the population, compared with 68% in unexpected noninfectious deaths (*P* = .001). There were significant differences noted for causes of death stratified by organ system; unexpected infectious deaths were more likely to be due to respiratory and intra-abdominal causes (*P* < .001 for each), whereas unexpected noninfectious deaths were more likely to be of cardiac etiology (*P* < .001). There were relatively more children and adolescents in the unexpected infectious death group (7%) than in the unexpected noninfectious death group (4%; *P* < .001).

Of the medical comorbid conditions, heart disease (52%), liver disease (31%), lung disease (29%), kidney disease (24%), and psychiatric conditions (24%) were the most prevalent in those with unexpected infectious deaths. Heart disease was the most common medical comorbid condition associated with all clinical syndromes, with the exception of endocarditis, for which liver disease was more prevalent. Toxicological analysis and coroner history revealed that a history of illicit drug use was present in 71 (17%) deaths, and acute drug intoxication was involved in 38 (9%). A history of smoking or alcohol use was present in 126 (30%) and 94 (23%) of deaths, respectively.

### Associated Clinical Syndromes

**Figure 1.**
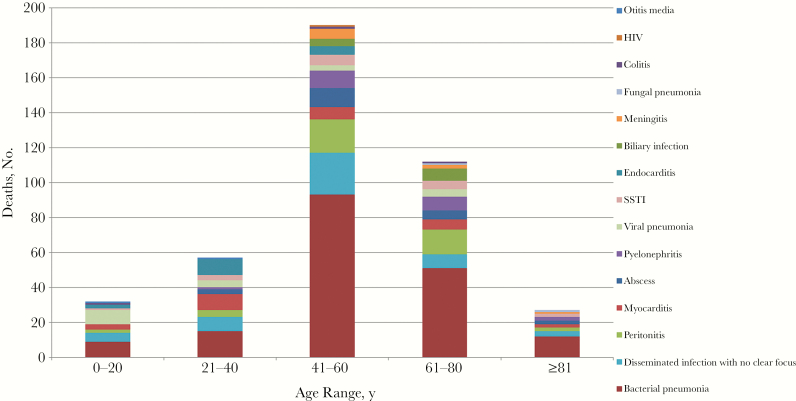
Cause of death by clinical syndrome and stratified by age group. Sample sizes for age groups are as follows: 0–20 years, n = 32; 21–40 years, n = 57; 41–60 years, n = 190; 61–80 years, n = 112; and ≥81 years, n = 27. Abbreviations: HIV, human immunodeficiency virus; SSTI, skin and soft-tissue infection.

The most common cause of death population-wide was bacterial pneumonia (43%) followed by disseminated infection with no clear focus (12%) and peritonitis (10%). *Streptococcus pneumoniae* (17 cases of 67 with positive cultures [25%]) and *S. aureus* (13 of 67 with positive cultures [19%]) were the most commonly isolated organisms in cases of bacterial pneumonia. When stratifying for age and sex, bacterial pneumonia was the most common through all subgroups (Figure 1). In the 0–20-year age cohort, viral pneumonia (25%) was relatively overrepresented compared with the general population, whereas the 21–40-year age cohort had overrepresentation of myocarditis and endocarditis as causes of death (16% each). The youngest median (IQR) ages noted for clinical syndromes were 28.0 (23–47.5) years for endocarditis, 32.0 (9–56) years for viral pneumonia, and 45.5 (29–64) years for myocarditis.

Heart disease (47% vs 43%; *P* = .03), lung disease (57% vs 43%; *P* < .001), smoking (56% vs 43%; *P* < .001), and alcohol use (62% vs 43%; *P* < .001) were more common among those dying of bacterial pneumonia compared with other infections. Both illicit drug use (17% vs 4%; *P* < .001) and liver disease (7% vs 4%; *P* = .02) were more commonly associated with death from endocarditis than with death from other infections. Psychiatric illness, kidney disease, cancer, and immunocompromised status were not significantly associated with any particular infection syndrome.

### Associated Pathogens

Causes of death were determined according to both the clinical and the pathogen, if applicable, associated with the syndrome (Table 2). Of the 418 deaths, we were able to identify pathogens responsible in 210 (50%). Pathogens were identified via a combination of antemortem cultures (30 deaths [7%]) and postmortem cultures (197 [47%]) with concordance seen in 14 of 16 cases (88%) in which both antemortem and postmortem cultures were isolated. *S. aureus* (35 deaths) was the most common individual pathogen associated with unexpected infectious death, followed by *S. pneumoniae* (30 deaths) and *Streptococcus pyogenes* (25 deaths). *S. aureus* was most often associated with bacterial pneumonia (37%), endocarditis (20%), and disseminated infection with no clear focus (20%). *S. pneumoniae* was predominantly associated with bacterial pneumonia (57%), followed by disseminated infection with no clear focus (23%) and meningitis (17%). 

**Table 2.** Most Common Clinical Syndromes and Pathogens Causing Unexpected Infectious Deaths in Descending Order of Frequency

Clinical Syndrome	Infectious Deaths, No. (%)
Bacterial pneumonia	180 (43.1)
Disseminated infection with no clear focus	48 (11.5)
Peritonitis	41 (9.8)
Myocarditis	26 (6.2)
Abscess	21 (5.0)
Pyelonephritis	21 (5.0)
Viral pneumonia	19 (4.5)
Skin/soft-tissue infection	17 (4.1)
Endocarditis	16 (3.8)
Biliary infection	11 (2.6)
Meningitis	10 (2.4)
Other	8 (1.9)
Pathogen	
*Staphylococcus aureus*	35 (8.4)
*Streptococcus pneumoniae*	30 (7.2)
*Streptococcus,* other^a^	29 (6.9)
*Streptococcus pyogenes*	25 (6.0)
*Klebsiella* spp.	23 (5.5)
*Escherichia coli*	19 (4.5)
Influenza A/B	16 (3.8)
Polymicrobial	14 (3.3)
*Proteus* spp.	7 (1.7)
Other	33 (7.9)
No pathogen isolated	208 (49.8)

^a^Including all *Streptococcus* spp. other than *S. pneumoniae* and *S. pyogenes*.

The most prevalent gram-negative organism was *Klebsiella* spp. (23 cases), which was associated with bacterial pneumonia (31%), pyelonephritis (22%), and peritonitis (13%). Of the 19 deaths from viral pneumonia, 13 were associated with an influenza virus, 3 were associated with noninfluenza viruses (1 death each with human metapneumovirus, parainfluenza 2, and rhinovirus), and 3 had no specific pathogen identified. The remaining deaths due to influenza virus were associated with myocarditis (2 cases with influenza growing in myocardial samples ) and bacterial pneumonia with a minor viral component (1 case with *S. aureus* the predominant organism). Among the 37 deaths in persons with a history of illicit drug use, *S. aureus* was the most common organism isolated (51%), followed by *S. pyogenes* (11%).

### Circumstances Around Death

**Figure 2.**
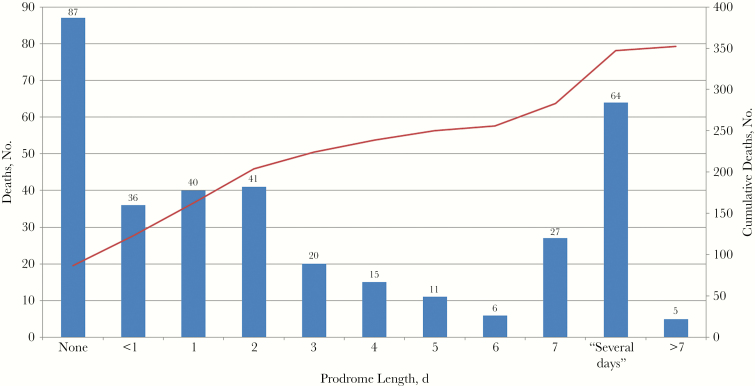
Prodrome length associated with 352 unexpected infectious deaths (length unknown in 66 deaths). Bar graphs represent number of deaths by length of prodrome; line graph, cumulative number of deaths.

The majority of unexpected infectious deaths were pronounced on the scene (n = 264 [63%]), with fewer persons pronounced dead on arrival to the emergency department (n = 73 [18%]) or after hospital admission (n = 81 [19%]). Within our population, 283 persons (68%) exhibited prodromal symptoms before death. Prodrome lengths were calculated for all persons who died, as well as stratified by clinical syndrome and pathogen (Figure 2 and Table 3). The median (IQR) prodrome length for the whole population was 1.0 (0.0–4.0) days. Persons with myocarditis and abscesses were found to have the shortest median prodromes, and those with meningitis the longest . Myocarditis, in particular, had the highest proportion of deaths without any prodrome (12 of 26 deaths). Influenza, *Klebsiella* spp., and *S. aureus* each had the shortest median prodrome, and *Escherichia coli* had the longest.

**Table 3.** Prodrome Lengths and Frequency of Recent Healthcare Contact by Clinical Syndromes and Pathogens, Sorted by Prodrome Length

Clinical Syndrome	Prodrome Length, Median (IQR), d	Recent Healthcare Contact, No. (%)
Myocarditis	0.0 (0.0–2.0)	31 (8)
Abscess	0.0 (0.0–2.0)	14 (3)
Disseminated infection with no clear focus	1.0 (0.0–2.0)	33 (16)
Bacterial pneumonia	1.0 (0.0–3.0)	20 (36)
Skin/soft-tissue infection	1.0 (0.0–3.0)	47 (8)
Pyelonephritis	1.0 (0.0–3.5)	43 (9)
Peritonitis	2.0 (0.0–3.0)	39 (16)
Biliary infection	2.0 (1.0–4.0)	55 (6)
Viral pneumonia	2.0 (0.0–5.5)	47 (9)
Endocarditis	3.0 (0.5–5.5)	25 (4)
Meningitis	4.0 (1.5–7.0)	30 (3)
Pathogens		
* Staphylococcus aureus*	1.0 (0.0–3.0)	29 (10)
* *Influenza A/B	1.0 (0.0–3.0)	44 (7)
* Klebsiella* spp.	1.0 (0.5–3.0)	26 (6)
* Streptococcus pneumoniae*	2.0 (0.0–3.5)	27 (8)
* Streptococcus pyogenes*	2.0 (1.0–3.0)	40 (10)
* Escherichia coli*	3.0 (0.0–6.0)	37 (7)

Abbreviation: IQR, interquartile range.

^a^Given the heterogeneity of the “*Streptococcus,* other” pathogen group, we excluded those persons from this analysis.

**Figure 3.**
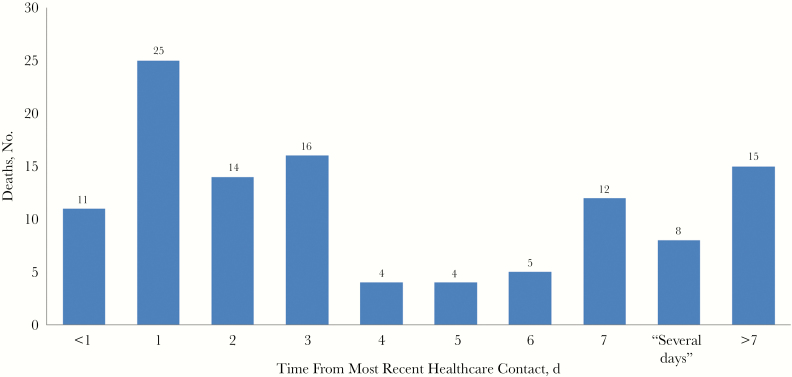
Time from the most recent healthcare contact, in days (n = 114, with the time frame unknown for 10 persons). The majority of deaths (291 of 418 [70%]) occurred in persons with no recent healthcare contact. (For the remaining 3 persons, it was unknown whether there was any healthcare contact, and they were excluded from this analysis.)

Of the 418 persons who died, only 124 (30%) had some form of healthcare contact in the preceding days before death (Figure 3 and Table 3). Among those with prior healthcare contact, the median (IQR) time lapse from healthcare contact to death was 3.0 (1.0–7.0) days.

## DISCUSSION

Our study demonstrates that infectious causes are responsible for 6% of all unexpected deaths within a large, heterogeneous population. The primary causes generally appear to be common, community-acquired sources of infections with the predominant cause being bacterial pneumonia, which represented 43% of unexpected infectious death cases. The causative organisms for unexpected infectious death within our autopsy cohort were primarily ubiquitous, gram-positive pathogens found in skin and oral flora. Medical comorbid conditions were common within our population, with 52% of those who died having underlying heart disease. Although a minority of persons who died presented with an opportunity for early intervention based on recent healthcare contact or a prolonged prodrome, the overall median symptom prodrome length of only 1.0 days suggests the need for upstream strategies designed to prevent these unexpected infectious deaths.

A majority of persons in this study died before exposure to the healthcare system, which presents a challenge in clinical care, particularly given the inability to provide appropriate antimicrobial therapy. The nonspecific and highly variable symptoms of sepsis may serve as barriers in presentation to a clinical setting and initiation of treatment. A large British cohort study identified infection to be responsible for 18% of all pediatric unexpected deaths, with a majority of infections being diagnosed only at autopsy as opposed to clinically before death [9]. The lack of clinical evidence of infection may be explained by the short prodrome length before death, particularly with causes such as myocarditis, which may result in death via sudden arrhythmia. 

Our study revealed that persons who died of biliary infection, skin and soft-tissue infection, viral pneumonia, or pyelonephritis had relatively more frequent recent healthcare contact than those who died of other infectious causes, which suggests potential opportunities for appropriate adequate antibiotic treatment, source control, and supportive care to prevent deaths in these patients. For other infectious syndromes with lower rates of healthcare contact, there may be benefit in public education encouraging people to seek care when they have early warning signs of severe illness. However, the overall low rate of healthcare contact (30%) and short prodrome lengths suggest that major gains in preventing these deaths would need to involve methods to prevent transmission of infections and host susceptibility rather than improvements in diagnosis and treatment.

The precise reason why some patients decline rapidly in the face of certain infectious diseases is not entirely clear. Within the infant population, the propensity for infection to cause unexpected death has been hypothesized to be related to an immature immune system producing an exaggerated anti-inflammatory response to mild infections [10]. The presence of bacterial superantigens has been well recognized, particularly associated in the well-described toxic shock syndrome with certain strains of staphylococcal and streptococcal infections [11]. *S. aureus* in particular has been associated with innate properties that allow it to evade immune system responses and present with fulminant infection, including necrotizing pneumonias [12, 13]. 

Given our study’s finding of bacterial pneumonia as the primary cause of unexpected infectious deaths, we believe this may relate to the simultaneous effects of hypoxic respiratory failure and sepsis-induced cardiovascular collapse. Respiratory infections are relatively common in the general population and have been shown to be a common source of unexpected infectious death in the neonate, pediatric, and young adult populations, although the current study is the first to confirm the predominance of respiratory infections across all age cohorts [9, 14–16]. The predominance of *S. aureus* within our study, particularly its relatively higher association with pneumonia compared with traditional community-acquired pneumonia pathogens (eg, *Haemophilus influenzae* and *Moraxella catarrhalis*), reaffirms the virulence of this organism and its role in rapid clinical decline in the community setting.

We believe that our study has several limitations. We probably undercaptured unexpected infectious deaths occurring in older, frail patients, given that performance of an autopsy required that the responsible coroner deem the death “unexpected.” Given most of the medical history and circumstances around death were reliant on coroner-obtained histories, there may have been variance in data collection, given the numerous coroners in the large jurisdiction of Ontario. For example, when nonspecific lengths of recent healthcare contact and prodromes were recorded, these data were excluded from our summary descriptive statistics. Similarly, given the numerous pathologists conducting autopsies, there was variability in the frequency of samples sent for microbiological data, resulting in likely underestimation of the maximum potential microbiological yield from autopsy. Our exclusion of hospital-acquired infections biases our results toward community-based organisms; however, an assessment into unexpected infectious death within hospitalized patients would require a similarly extensive study given major differences in patient populations and microbiology. Given that ethics approval was applicable only to the coroner report and autopsy findings, we were unable to explore specific demographic and sociological factors, such as distance from a healthcare facility, social living situation, and income, which may affect timely access to healthcare.

Overall, our study findings show that infectious diseases have the potential for rapid and fulminant clinical decline in a subset of the population. The most common cause of unexpected infectious death within our population was bacterial pneumonia and, predominantly, gram-positive bacteria. We believe that our study highlights potential opportunities for intervention, because a number of persons who died had recent healthcare contact before death, and deaths were most frequently caused by common community infections and pathogens. 

Given that the majority of deaths in our study were declared on the scene, strategies to improve timely diagnosis and treatment, such as paramedic-guided antimicrobial therapy, will unfortunately not prevent most of these deaths. Ideally, there should be a continual focus toward upstream strategies, such as public education regarding warning signs of severe infection, promoting early access to urgent care facilities and phone triage systems, and preventing infection transmission through vaccine development and implementation. Further research will be required to explore specific host-pathogen interactions to elucidate why certain persons die quickly when faced with an infection while others are more resilient.
